# A Cluster of Ocular Syphilis Cases with a Common Sex Partner — Southwest Michigan, 2022

**DOI:** 10.15585/mmwr.mm7247a1

**Published:** 2023-11-24

**Authors:** William D. Nettleton, James B. Kent, Karen Lightheart, Jill C. Diesel

**Affiliations:** ^1^Kalamazoo County Health and Community Services Department, Kalamazoo, Michigan; ^2^Department of Family and Community Medicine, Western Michigan University Homer Stryker M.D. School of Medicine, Kalamazoo, Michigan; ^3^Michigan Department of Health & Human Services; ^4^Division of STD Prevention, National Center for HIV, Viral Hepatitis, STD, and TB Prevention, CDC.

**Summary**

**What is already known about this topic?**

Untreated syphilis can lead to rare manifestations of ocular syphilis, otosyphilis, and neurosyphilis. Prompt diagnosis and treatment of syphilis can prevent systemic complications.

**What is added by this report?**

A cluster of five cases of ocular syphilis in women with a common male sex partner was identified in Michigan, suggesting that an unidentified *Treponema pallidum* strain might have been a risk factor for developing systemic manifestations of syphilis.

**What are the implications for public health practice?**

Maintaining a high index of clinical suspicion and obtaining a thorough sexual history are critical to diagnosing ocular syphilis, otosyphilis, and neurosyphilis. Coordination of disease surveillance with disease intervention specialist case investigation, partner notification, and treatment referral can interrupt syphilis transmission.

## Abstract

Untreated syphilis can lead to ocular syphilis, otosyphilis, and neurosyphilis, conditions resulting from *Treponema* *pallidum* infection of the eye, inner ear, or central nervous system. During March–July 2022, Michigan public health officials identified a cluster of ocular syphilis cases. The public health response included case investigation, partner notification, dissemination of health alerts, patient referral to a public health clinic for diagnosis and treatment, hospital care coordination, and specimen collection for *T. pallidum* molecular typing. Five cases occurred among southwest Michigan women, all of whom had the same male sex partner. The women were aged 40–60 years, HIV-negative, and identified as non-Hispanic White race; the disease was staged as early syphilis, and all patients were hospitalized and treated with intravenous penicillin. The common male sex partner was determined to have early latent syphilis and never developed ocular syphilis. No additional transmission was identified after the common male partner’s treatment. Due to lack of genetic material in limited specimens, syphilis molecular typing was not possible. A common heterosexual partner in an ocular syphilis cluster has not been previously documented and suggests that an unidentified strain of *T. pallidum* might have been associated with increased risk for systemic manifestations of syphilis. A high index of clinical suspicion and thorough sexual history are critical to diagnosing ocular syphilis, otosyphilis, and neurosyphilis. Coordination of disease surveillance with disease intervention specialist investigation and treatment referral can interrupt syphilis transmission.

## Investigation and Results

In Michigan, all reactive syphilis laboratory test results are routinely reported to the Michigan Disease Surveillance System (MDSS). Syphilis case investigation and contact tracing are centralized to the Michigan Department of Health & Human Services (MDHHS), whereas treatment and care are coordinated by local public health departments and health care facilities. On April 21, 2022, a local public health physician at Kalamazoo County Health and Community Services Department (KCHCSD) alerted MDHHS that two cases of ocular syphilis had been identified during the previous 5 weeks in two hospitalized women (patient A and patient B) who were from the same geographic area (Figure). An epidemiologic link was established between patients A and B when a common male sex partner was identified. MDHHS and KCHCSD, which includes a sexual health clinic with comprehensive testing, treatment, and counseling services, coordinated response and investigation of the patients in the cluster. Molecular typing to investigate the genetic strain of syphilis was not possible because of a lack of genetic material in the limited available specimens. This activity was reviewed by CDC, deemed not research, and was conducted consistent with applicable federal law and CDC policy.*

**FIGURE:**
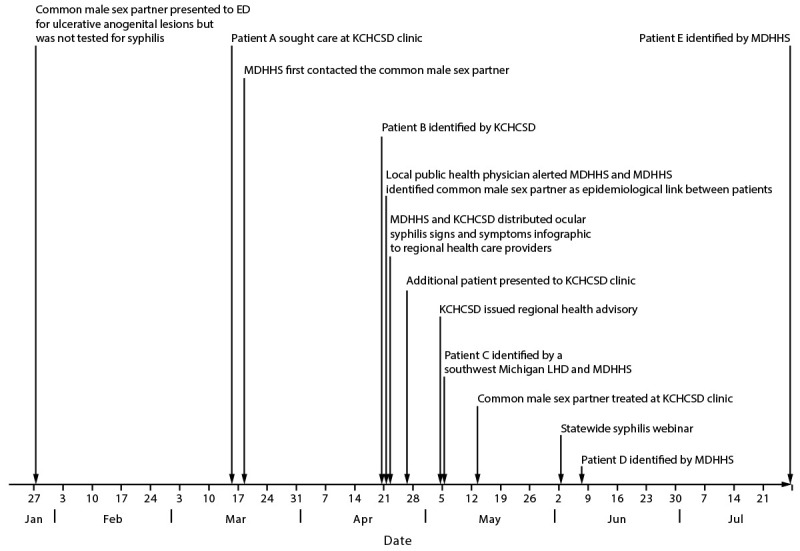
Investigation and response timeline of an ocular syphilis cluster with a common sex partner — southwest Michigan, 2022* <Figure_Large></Figure_Large> **Abbreviations:** ED = emergency department; KCHCSD = Kalamazoo County Health and Community Services Department; LHD = local health department; MDHHS = Michigan Department of Health & Human Services. * Patients D and E were exposed to the common male sex partner before his treatment.

### Clinical and Epidemiologic Characteristics of Cluster Patients

Among all five women eventually identified in the cluster, prophylactic treatment was offered to every sex partner for whom contact information was available. Each of the five women in the cluster lived in a different southwest Michigan county and were aged 40–60 years (mean = 49.0 years) and identified as White race. All were hospitalized and received intravenous penicillin treatment (Table 1). All were HIV-negative, and none reported drug use or transactional sex. Reported routes of sexual exposure among the five women included anal (40%), oral (40%), and vaginal (100%). 

**TABLE 1:** Staging, clinical manifestations, and outcomes of a cluster of ocular syphilis patients — southwest Michigan, 2022

Patient*	Syphilis stage^†^	Ocular manifestation^†^	Neurologic manifestation^†^	Syphilis serologies	CSF result	Hospitalization	Treatment^§^
A	Primary	Likely	No	TPPA reactive USR 1:32	Negative	3 days	IV penicillin x 14 days
B	Secondary	Likely	Verified	MIA reactive USR 1:64	VDRL 1:16	6 days	IV penicillin x 14 days
C	Secondary	Possible	Verified	TPPA reactive RPR 1:512	VDRL 1:8	6 days	IV penicillin x 14 days
D	Secondary	Likely	No	TPPA reactive RPR 1:256	Test not conducted	4 days	IV penicillin x 14 days
E	Early latent	Likely	Likely	IgG positive USR 1:512	VDRL 1:2	21 days	IV penicillin x 14 days
Common male sex partner	Early latent	NA	NA	IgG positive USR 1:64	NA	None	IM penicillin once

**Abbreviations:** CSF = cerebrospinal fluid; IgG = immunoglobulin G antibody; IM = intramuscular; IV = intravenous; MIA = multiplex immunoassay; NA = not applicable; RPR = rapid plasma regain; TPPA = *Treponema* *pallidum* particle agglutination; USR = unheated serum regain; VDRL = venereal disease research laboratory test.

* A sixth patient was determined to be unrelated to the cluster through case investigation and is not included.

^†^ Syphilis staging and ocular and neurologic manifestations as defined by the 2018 Council of State and Territorial Epidemiologists syphilis case definition.

^§^ Outpatient IV penicillin treatment, often by continuous infusion pump, enabled IV treatment duration to exceed duration of hospitalization.

Patient A was referred to KCHCSD in March 2022 by an ophthalmologist for a reactive treponemal antibody test result. Patient A noted blurred vision, fear of blindness, and no improvement in genital lesions with valacylocvir, which the patient had been taking for presumed recurrent herpes simplex virus infection. She received a diagnosis of primary and ocular syphilis, and care was coordinated with hospital A for treatment. An interview identified a recent male sex partner whom patient A had met online. Patient A stated she had no other sex partners during the previous 12 months.

Patient B was identified by KCHCSD’s communicable disease surveillance team in April 2022, having been admitted to hospital A for neurosyphilis. Before admission, she reported headache, mild hearing loss, and worsening blurry vision and double vision for 4 weeks; she had been treated in ambulatory care settings with amoxicillin, oral and intranasal steroids, and antiinflammatory medications, and was referred to an emergency department by an ophthalmologist who noted cranial nerve abnormalities. Patient B named the same recent sex partner named by patient A; patient B also met this partner online. A second named partner of patient B was contacted and received a negative syphilis test result.

Patient C received a reactive syphilis test result and was reported by a clinician to a local health department in southwest Michigan in May 2022. Patient C had a full body rash and peeling skin on the palms of her hands; she reported spots drifting through her field of vision (floaters) and photophobia. The patient was prescribed oral steroids, evaluated by an ophthalmologist, underwent a magnetic resonance imaging study of the brain, and was treated with 1 dose of intramuscular penicillin. MDHHS disease intervention specialists^†^ and a local public health physician coordinated inpatient evaluation at hospital A, where the patient was found to have cranial nerve abnormalities. Patient C named the same male sex partner named by patients A and B; patient C also met this partner online. After follow-up by disease intervention specialists, patient C named three additional sex partners, and reported that each of these partners told her that they had received a negative syphilis test result.

Patient D received a diagnosis of ocular syphilis from an ophthalmologist in June 2022, after referral to hospital B for worsening vision. During the preceding months, patient D had experienced genital sores and a rash on her hands and abdomen, for which steroids were prescribed. Patient D named the same male sex partner named by patients A, B, and C as a sexual contact during January 2022. Two other sex partners of patient D received negative syphilis test results.

Patient E sought evaluation at hospital B’s ophthalmology clinic in May 2022 for visual floaters, seeing flashing lights, and worsening vision after cataract surgery 3 months earlier. She received a reactive treponemal test result, but a nontreponemal test was not performed. Since only a fraction of reactive treponemal test results identify active infections that can be transmitted to others, MDHHS protocols defer certain investigations until additional results are reported. In July, patient E was admitted to hospital B with neurosyphilis and ocular syphilis. A reactive cerebrospinal fluid venereal disease research laboratory result triggered an MDHHS investigation. During February–April 2022, patient E had sexual contact with the same male partner reported by patients A, B, C, and D. Two other partners of patient E were unnamed; therefore, they could not be contacted.

### Common Male Sex Partner

The common male sex partner of patients A–E was contacted by telephone and text message on multiple occasions by MDHHS disease intervention specialists during March–May 2022. He provided limited information, stated that he had traveled out of Michigan, and did not attend a scheduled appointment for evaluation in April. In May 2022, after patient C named the same male partner as patients A and B, a local public health physician reviewed the common partner's electronic medical records and discovered that he had sought care at hospital A's emergency department in January 2022 with ulcerative penile and anal lesions. At that time, he was treated with acyclovir for presumed herpes simplex virus infection, a nucleic acid amplification test for herpes simplex virus was negative, and no syphilis serology tests were ordered. After a MDHHS disease intervention specialist renewed contact with him, the common partner scheduled and kept an appointment at KCHCSD in May 2022. Upon evaluation, no signs or symptoms of syphilis were found, and he reported no visual or hearing impairment. On sexual history, he reported having multiple female sex partners during the previous 12 months, but he declined to disclose their identities; he reported no male or transgender sexual contact. He received a diagnosis of laboratory-confirmed early latent syphilis and was treated with 1 dose of intramuscular penicillin. In follow-up interviews, both patient A and patient B stated that the male sex partner had a sore on his penis in January 2022.

### Additional Ocular Syphilis Patients

Public health officials used MDSS to compare patients in this ocular syphilis cluster to other patients with ocular syphilis occurring during a similar time frame (Table 2). Among 43 ocular syphilis patients who were not part of the cluster, 19% were HIV-positive, 2% reported injection drug use, and 7% reported transactional sex.

**TABLE 2:** Demographic and clinical characteristics for ocular syphilis cluster patients and other ocular syphilis patients from a similar time frame — southwest Michigan, 2022

Characteristic	No. (%)	
	Patients in cluster, southwest Michigan	Patients not in cluster, Michigan
**Time frame**	March–July	January–August
**Total (no.)**	5 (100)*	43 (100)
**Sex**		
Men	0 (—)	32 (74)
Women	5 (100)	11 (26)
**Age range, yrs (mean)^†^**	40–60 (49.0)	22–75 (43.6)
**Age group, yrs**		
20–29	0 (—)	5 (12)
30–39	0 (—)	14 (33)
40–49	3 (60)	12 (28)
50–59	2 (40)	5 (12)
≥60	0 (—)	7 (16)
**Race**		
Asian	0 (—)	1 (2)
Black or African American	0 (—)	11 (26)
White	5 (100)	26 (60)
Other race	0 (—)	4 (9)
Unknown	0 (—)	1 (2)
**Hispanic ethnicity**		
Non-Hispanic	5 (100)	39 (91)
Hispanic	0 (—)	1 (2)
Unknown	0 (—)	3 (7)
**Sexual behavior**		
Heterosexual men	0 (—)	9 (21)
Heterosexual women	5 (100)	8 (19)
Men who have sex with men	0 (—)	11 (26)
Sex with anonymous partner	0 (—)	10 (23)
Sex without a condom	5 (100)	26 (60)
Met partner on social media	5 (100)	8 (19)
**Syphilis staging and manifestation and comorbidity**		
Primary	1 (20)	2 (5)
Secondary	3 (60)	7 (16)
Early	1 (20)	5 (12)
Late latent	0 (—)	29 (67)
Neurosyphilis codiagnosis	3 (60)	11 (26)
STI comorbidity and history		
HIV-positive	0 (—)	8 (19)
Previously documented STI CT, NG, or syphilis	1 (20)	13 (30)
**Residence** ^§^		
Southeast Michigan	0 (—)	22 (51)
Southwest Michigan^¶^	5 (100)	5 (12)
Allegan County	1 (20)	1 (2)
Berrien County	0 (—)	1 (2)
Branch County	1 (20)	0 (—)
Kalamazoo County	1 (20)	3 (7)
Saint Joseph County	1 (20)	0 (—)
Van Buren County	1 (20)	0 (—)
**Other risk factors**		
Reported injection drug use	0 (—)	1 (2)
Unhoused	1 (20)	Unknown
Transactional sex	0 (—)	3 (7)

**Abbreviations**: CT = *Chlamydia trachomatis*; NG = *Neisseria gonorrhea*; STI = sexually transmitted infection.

* A sixth patient was determined to be unrelated to the cluster through case investigation and is not included.

^†^ Age ranges were rounded to the nearest 10 years to prioritize privacy; age mean for patients in cluster did not use rounded age values.

^§^ Southeast Michigan includes Macomb, Oakland, and Wayne counties; for this analysis, southwest Michigan includes Allegan, Barry, Berrien, Branch, Calhoun, Cass, Eaton, Kalamazoo, Saint Joseph, and Van Buren counties.

^¶^ Ocular syphilis cluster cases occurred in five of the 10 total southwest Michigan counties.

A sixth patient, identified in April 2022, was determined to be unrelated to the cluster because no sexual link to the five other ocular syphilis cases or the common sex partner was found. This male patient sought treatment at KCHCSD, and received a diagnosis of secondary syphilis with ocular and otic manifestations, and was admitted to hospital A. A cerebrospinal fluid nontrepenomal antibody test was reactive, and the patient was treated with 14 days of intravenous penicillin. He named two male sex partners, which did not include the same common male sex partner reported by the five female patients. 

### Public Health Response

In late April 2022, MDHHS and KCHCSD distributed an infographic to Michigan health care providers via local and state public health sexually transmitted infection email distribution lists regarding signs and symptoms of ocular syphilis, otosyphilis, and neurosyphilis. The MDHHS infographic prompted one physician to notify the sixth patient that his symptoms might indicate ocular syphilis; this resulted in the patient’s seeking medical evaluation. In early May 2022, KCHCSD issued a health advisory to area clinicians and to surrounding counties via the Michigan Health Alert Network describing 1) the ocular syphilis cases to date; 2) signs and symptoms of ocular syphilis, otosyphilis, and neurosyphilis; 3) recommendations for obtaining thorough sexual histories, conducting medical evaluations, reporting cases to public health, and consulting with specialists; and 4) recommended treatment options. In early June 2022, KCHCSD, MDHHS, and the New York City STD/HIV Training and Prevention Center presented a training webinar on syphilis diagnosis and treatment, highlighting the southwest Michigan ocular syphilis cluster to county health department nurses, physicians, and sexually transmitted infection staff members from across Michigan.

## Discussion

The association between five women with ocular manifestations of syphilis and a common male sex partner is an unusual occurrence and suggests that an unidentified strain of *T. pallidum* might have been associated with increased risk for systemic manifestations of syphilis in these patients. This ocular syphilis cluster is the first documented with epidemiologic linkage among cases attributable to heterosexual transmission. In 2019, a study of 41,187 syphilis cases from 16 jurisdictions with complete reporting, including Michigan, found that incidence of systemic manifestations were rare (neurosyphilis, 1.1%; ocular syphilis, 1.1%; and otosyphilis, 0.4%) (*1*). A cluster of ocular syphilis was reported in Seattle in 2015 among four men who have sex with men, three of whom were HIV-positive and two of whom were sex partners (*2*). Among 139 suspected ocular syphilis cases with partner data from four U.S. jurisdictions during 2014–2015, none of the partners had ocular syphilis (*3*).

Although ocular and neurosyphilis can occur at any stage of syphilis, a 2019 U.S. prevalence estimate found that these clinical manifestations occurred more commonly during late-stage syphilis, and were most prevalent among persons aged ≥65 years and those reporting injection drug use (*1*). In contrast, among cases in the current reported cluster, all patients had early-stage disease, and were aged 40–60 years, and none reported injection drug use or transactional sex. Although approximately 40% of patients with ocular or neurologic manifestations of syphilis in the 2019 prevalence estimate were HIV-negative, all patients in this cluster were HIV-negative.

The rate of primary and secondary syphilis in Michigan increased from 3.8 per 100,000 persons in 2016, predominantly in southeast Michigan, to 9.7 in 2022, with increasing incidence in southwest Michigan. Although the majority of primary and secondary syphilis cases in Michigan in 2022 occurred in men (77%), and 39% were in men who have sex with men, the proportion of cases occurring in women increased from 9% in 2016 to 23% in 2022. The rate of primary and secondary syphilis among women in Michigan has increased from 2016 to 2022 (from 0.3 to 2.2 per 100,000 among White women and from 2.6 to 15.5 per 100,000 among Black or African American [Black] women).^§^

Differential ascertainment bias might contribute to more frequent identification of ocular syphilis, otosyphilis, or neurosyphilis among White persons than among those who identify as Black or Hispanic in the United States (*1*). Although all five patients in the observed cluster were non-Hispanic White women, differential ascertainment bias and rising syphilis incidence among Michigan women do not explain the finding of a common sex partner. Michigan has not changed case-based syphilis surveillance reporting methodology, but did implement a systemic manifestation checklist and algorithm in 2020 to improve precision in classifying ocular, otic, and neurologic manifestations, to align with 2018 Council of State and Territorial Epidemiologists’ syphilis surveillance definitions.^¶^

Sexually transmitted infection transmission depends upon biologic host and pathogen factors, individual and population risk behaviors, shared social networks, and disease prevalence (*4**,**5*). Recommended treatment reduces the duration of infectiousness, thereby interrupting transmission (*4*). Disease clusters might be explained by strain-specific pathogen factors or shared host susceptibility characteristics; however, no shared host susceptibility characteristics were identified among patients in this cluster. In addition, no disease transmission linked to the cluster was identified after treatment of the male sex partner, and no ocular syphilis patients with sexual linkage to others who also developed ocular syphilis have since been identified in Michigan. These limited observations suggest the possibility that a specific strain of *T. pallidum* might have been associated with ocular and neurosyphilis among the observed patients and ceased to circulate after these patients and their common partner were treated. However, without cluster-specific or wider geographic *T. pallidum* molecular typing surveillance, this hypothesis cannot be confirmed. Molecular typing studies linking ocular or neurologic manifestations to specific *T. pallidum* strains produced mixed findings (*6*,*7*). Successful *T. pallidum* DNA detection by nucleic acid amplification is most feasible from a primary ulcer or moist secondary lesion (*8*,*9*), but in this cluster, only patient A had primary syphilis at the time of diagnosis. Optimized specimen collection procedures and development of standardized *T. pallidum* DNA detection techniques from secondary lesions, serum, whole blood, and cerebrospinal fluid might enhance future evaluation of oculo- or neurotropic potential of *T. pallidum* strains (*9*).

A local health department with a sexual health clinic, public health physician, and integrated communicable disease surveillance team facilitated initial clinical diagnosis of cases, hospital care coordination, communication to state disease surveillance teams, and treatment of the common sex partner. Case investigation by state disease intervention specialists and partner notification led to the identification of the common sex partner and facilitated treatment referral, resulting in interruption of disease transmission across county jurisdictions. 

### Implications for Public Health Practice

Coordination of disease surveillance with disease intervention specialist investigation and treatment referral can interrupt syphilis transmission. Maintaining a high index of clinical suspicion and obtaining a thorough sexual history are critical for diagnosis of ocular syphilis, otosyphilis, and neurosyphilis in all clinical settings.** Prompt diagnosis and treatment of syphilis can prevent systemic complications, including permanent visual or hearing loss. Persons at risk for syphilis should be evaluated for neurologic, visual, and auditory symptoms; likewise, a careful neurologic examination and neurologic, visual, and auditory symptom evaluation should be conducted among persons with syphilis infection. An immediate ophthalmologic evaluation should be facilitated for persons with syphilis and ocular complaints. Any cranial nerve dysfunction should prompt a lumbar puncture and cerebrospinal fluid evaluation before treatment, if possible^.^^††^ The CDC 2021 Sexually Transmitted Infections Treatment Guidelines offer recommendations for treatment of ocular syphilis, otosyphilis, and neurosyphilis (*10*).

## Footnotes

* 45 C.F.R. part 46 102(1)(2), 21 C.F.R. part 56; 42 U.S.C. Sect. 241(d); 5 U.S.C. Sect. 552a; 44 U.S.C. Sect. 3501 et seq.

† https://www.cdc.gov/std/projects/disease-intervention/default.html

§
https://www.michigan.gov/mdhhs/-/media/Project/Websites/mdhhs/Keeping-Michigan-Healthy/HIVSTI/Data-and-Statistics/2022/2013-2022-STI-Trends-in-Michigan-Tables-Summary.pdf

¶ https://cdn.ymaws.com/www.cste.org/resource/resmgr/2017PS/2017PSFinal/17-ID-11.pdf

** https://www.cdc.gov/std/syphilis/stdfact-syphilis-detailed.htm

††
https://www.cdc.gov/std/syphilis/neuro-ocular-oto.htm
